# Use of *Anthracophyllum discolor* and *Stereum hirsutum* as a Suitable Strategy for Delignification and Phenolic Removal of Olive Mill Solid Waste

**DOI:** 10.3390/foods11111587

**Published:** 2022-05-28

**Authors:** Viviana Benavides, Fernanda Pinto-Ibieta, Antonio Serrano, Olga Rubilar, Gustavo Ciudad

**Affiliations:** 1 Programa de Doctorado en Ciencias de Recursos Naturales, Facultad de Ingeniería y Ciencias, Universidad de La Frontera, Casilla 54-D, Temuco 4780000, Chile; d.benavides01@ufromail.cl; 2 Departamento de Ingeniería Química, Facultad de Ingeniería y Ciencias, Universidad de La Frontera, Avenida Francisco Salazar #01145, Casilla 54-D, Temuco 4780000, Chile; f.pinto05@ufromail.cl (F.P.-I.); olga.rubilar@ufrontera.cl (O.R.); 3 Departamento de Procesos Industriales, Facultad de Ingeniería, Universidad Católica de Temuco, Casilla 15-D, Temuco 4780000, Chile; 4 Departamento de Microbiología, Facultad de Farmacia, Campus Universitario de Cartuja s/n, Universidad de Granada, 18011 Granada, Spain; antonio.serrano@ugr.es; 5 Instituto de Investigación del Agua, Universidad de Granada, 18071 Granada, Spain; 6 Scientific and Technological Bioresources Nucleus (BIOREN), Universidad de La Frontera, Avenida Francisco Salazar #01145, Casilla 54-D, Temuco 4780000, Chile; 7 Instituto del Medio Ambiente (IMA), Universidad de La Frontera, Avenida Francisco Salazar #01145, Casilla 54-D, Temuco 4780000, Chile

**Keywords:** white-rot fungi, OMSW, ligninolytic enzyme, pretreatment, agroindustrial waste valorization

## Abstract

This study evaluated the use of the white-rot fungi (WRF) *Anthracophyllum discolor* and *Stereum hirsutum* as a biological pretreatment for olive mill solid mill waste (OMSW). The WRF strains proposed were added directly to OMSW. The assays consisted of determining the need to add supplementary nutrients, an exogenous carbon source or use agitation systems, and evaluating WRF growth, enzyme activity, phenolic compound removal and lignin degradation. The highest ligninolytic enzyme activity was found at day 10, reaching 176.7 U/L of manganese-independent peroxidase (MniP) produced by *A. discolor*, and the highest phenolic removal (more than 80% with both strains) was reached after 24 days of incubation. The confocal laser scanning microscopy analysis (CLSM) confirmed lignin degradation through the drop in lignin relative fluorescence units (RFU) from 3967 for untreated OMSW to 235 and 221 RFU, showing a lignin relative degradation of 94.1% and 94.4% after 24 days of treatment by *A. discolor* and *S. hirsutum,* respectively. The results demonstrate for the first time that *A. discolor* and *S. hirsutum* were able to degrade lignin and remove phenolic compounds from OMSW using this as the sole substrate without adding other nutrients or using agitation systems. This work indicates that it could be possible to design an in situ pretreatment of the valorization of OMSW, avoiding complex systems or transportation. In this sense, future research under non-sterile conditions is needed to evaluate the competition of WRF with other microorganisms present in the OMSW. The main drawbacks of this work are associated with both the low reaction time and the water addition. However, OMSW is seasonal waste produced in one season per year, being stored for a long time. In terms of water addition, the necessary optimization will be addressed in future research.

## 1. Introduction

Every year, close to 1000 Gt of lignocellulosic biomass is generated worldwide, including wheat straw, sugarcane bagasse, corn stalks, and others [1,2]. This huge volume of biomass can be used as suitable feedstocks in biorefineries to obtain added-value products derived from the polysaccharide fraction, including carbohydrate-enriched ruminant feed, chemical compounds, and biofuels [3,4,5,6]. However, the molecular structure of lignin and its strong association with cellulose and hemicellulose strongly limit the acquisition of these added-value products [7,8]. To resolve this challenge, enzymatic pretreatments have been proposed to enhance the use of lignocellulosic biomass as feedstock in subsequent bioprocesses [9]. Some authors have reported the direct use of ligninolytic enzymes for the reduction of the lignin content in different lignocellulosic wastes [10,11,12]. Recently, the use of white-rot fungi (WRF) has been proposed as an alternative to the direct application of pure enzymes to delignify lignocellulosic residues [13,14,15]. WRF can produce extracellular oxidative enzymes such as laccase (Lac), manganese peroxidase (MnP) and manganese-independent peroxidase (MniP), which have been reported as being involved in the degradation of lignocellulosic fibers [16,17]. In this sense, different WRF strains have been proposed as pretreatments for different lignocellulosic residues, such as sweet sorghum bagasse, radiata pine and wheat straw, among others [15,18,19].

During olive oil extraction in two phase processes, a semi-solid byproduct called olive mill solid waste (OMSW) is generated, presenting a variable water content of 60–95% [20]. The incorrect management of OMSW has several deleterious environmental effects, such as coloration of natural waters, toxicity to aquatic life, degradation of soil quality by inhibiting plant germination and growth, phytotoxicity, and the generation of nuisance odors [21,22]. OMSW is composed of olive husk, olive pulp, and olive vegetation water, resulting in a substrate rich in lignocellulosic fibers and polyphenols [23,24]. Polyphenols in OMSW create a high antimicrobial activity that can hamper the valorization of this biomass through microbe-mediated processes [25]. In this context, few studies have described the use of WRF for the pretreatment and further valorization of OMSW. According to the literature, the effectiveness of WRF for phenol removal is strongly dependent on the selected fungi. For example, phenol removal in OMSW varied between 50% and 85% using *Phanerochaete chrysosporium* [17] and *Phlebia* sp. [26], respectively. More recently, *Pleurotus citrinopileatus* and *Irpex lacteus* appeared as potent degraders of olive mill wastewater, while simultaneously producing biotechnologically relevant enzymes [27]. These co-generated enzymes could be used to degrade other lignocellulosic waste or to bioremediate soil contaminated with pentachlorophenol [27,28,29]. Therefore, the application of WRF to OMSW might not only result in the phenolic removal of the biomass, but also in the acquisition of valuable ligninolytic enzymes. In particular, *Anthracophyllum discolor* and *Stereum hirsutum* are Chilean native WRF isolated from temperate forests in southern Chile that have been reported to generate lignocellulosic enzymes such as MnP and LiP [30]. Both WRF have been used in pentachlorophenol removal from soil [29] and used as pretreatment of agroindustrial waste such as wheat straw [1]. Therefore, *A. discolor* and *S. hirsutum* could be suitable strains for the pretreatment of lignocellulosic waste like OMSW. In this context, the novelties of this work are: (a) *A. discolor* and *S. hirsutum* have not been previously evaluated in this waste, (b) there are few studies oriented to OMSW treatment using WRF, with most focusing on olive mill wastewater treatment from three phase processes, all with other WRF strains [16,17,27], and (c) this work proposes applying the strains directly, in the static condition (without agitation or addition of an additional carbon source or nutrient); thus, the number of steps in this process is minimal and does not require any additional feedstock to culture the WRF. The use of local fungal strains could be very interesting because it would avoid the introduction of foreign organisms in the ecosystems, which would potentially result in several environmental impacts [31]. Furthermore, local fungal strains would already be adapted to the environmental conditions of the area where the fungal treatment will be carried out, which would be very desirable for the subsequent scalation and industrial implementation of the process. Therefore, the aim in this work was to evaluate the direct application of *A. discolor* and *S. hirsutum* as a biological pretreatment for OMSW.

## 2. Materials and Methods

### 2.1. Microorganisms

The fungal strains used were *A. discolor* Sp4 and *S. hirsutum*, both isolated from decayed wood in the temperate forests of southern Chile belonging to the Culture Collection of the Environmental Nanobiotechnology Laboratory at the Universidad de La Frontera, Chile. The fungi were stored at 4 °C in Petri dishes with potato dextrose agar (PDA) medium until use. The culture medium was prepared using 39 g of PDA in 1 L of distilled water and sterilized in an autoclave for 20 min at 121 °C. Then, the strains were activated in PDA medium using one disk of 6 mm as the initial inoculum and incubated at 25 °C for 7 days for the assays.

### 2.2. Olive Mill Solid Waste (OMSW)

OMSW was collected from the olive mill “Olivares de Quepu”. The olive tree variety is Arbequina for extra virgin olive oil production. OMSW was stored in self-sealing bags for refrigerated storage at −20 °C. For all tests, OMSW was used directly, without applying any pretreatment such as grinding or sieving.

Characterization of OMSW included oxygen demand (COD), total solids (TS), volatile solids (VS), pH, total nitrogen (TN), and ammonia nitrogen (N-ammonia), all measured according to standard methods [30]. Cu, Fe, and Mn present in OMSW were measured by EPA method 6020A [31]. NO_2_- and NO_3_- anions were measured by ion chromatography [31].

Total phenol content in the liquid and solid phases were quantified by spectrophotometry with a gallic acid (GA) calibration curve, using the Folin-Ciocalteu method, expressing the results as mg GA/L and mg GA/100 g [32]. For the determination of total phenol content in solid phases, previous extraction was carried out using 1 g of OMSW and 1 mL of methanol/water (80:20 *v*/*v*). The mixture was stirred for 1 min in a vortex apparatus and centrifuged at 1200 G for 10 min. The methanol layer was separated and the extraction was repeated four times [32]. For phenolic profile determination, samples were treated with a water/methanol/formic acid solution (24:25:1) and sonicated. They were allowed to stand for 24 h to sonicate again, centrifuged and finally filtered for further analysis. A Hitachi Primaide high-performance liquid chromatograph coupled to a diode array detector (HPLC-DAD) was used, equipped with a Kromasil ^®^ C18 column [33].

The ash, total extractives, lignin, hemicellulose, and cellulose contents were measured by the Klason lignin method (TAPPI T 222 om-02) [30]. Lipids were analyzed by the soxhlet extraction method described in the standard methods of the American Public Health Association APHA [34].

### 2.3. Qualitative Detection of Lignocellulolytic Enzymes

Qualitative discoloration tests with Remazol Brilliant Blue R (RBBR) and staining with 2,2′-azino-bis (3-ethylbenzothiazolin-6-sulfonic acid) (ABTS) (Calbiochem) were performed. PDA culture medium enriched with sterile glucose aqueous solution 20% (*w*/*v*) and ABTS was used to qualitatively determine the presence of oxidase enzymes (laccases) in fungal strains and RBBR to determine peroxidase enzymes (manganese peroxidase and lignin peroxidase) [34]. The tests were performed in triplicate in Petri dishes, using uninoculated plates as controls. One agar disk (6 mm diameter) of active mycelium from a 7-day-old culture in PDA medium was inoculated in Petri dishes and then incubated at 25 °C in darkness. The green halo in culture medium with ABTS and the blue to yellow discoloration in culture medium with RBBR were periodically evaluated for 4, 7, and 14 days. The discoloration and coloration scale proposed by Tortella et al. [30] was used in this assay.

### 2.4. Mycelial Growth Assays

The mycelial growth was evaluated in three different solid mediums: (a) PDA medium, (b) OMSW, and (c) OMSW+PDA. In the experiment, 20 g of OMSW were distributed on Petri dishes and in the combined conditions, OMSW was added to solidified PDA. OMSW (62% moisture content) was sterilized under the same conditions. Then, Petri dishes were inoculated with one agar disk (6 mm diameter) of active mycelium from a 7-day-old culture in PDA medium and were incubated at 25 °C for 12 days or until the dish was covered completely by the fungi. The experiment was carried out in triplicate. The mycelial growth was evaluated every 24 h for 12 days by photographic registration, so that the current limit of the mycelia growth could be visualized. The public domain program ImageJ v.1.52a was used for image processing.

The kinetics of the growth of *A. discolor* and *S. hirsutum* were evaluated by applying the modified Gompertz equation to the experimental data of mycelial growth monitored during the experimental period (Equation (1)). The application of the modified Gompertz equation was previously reported for the growth of *Fusarium verticillioides* and *Rhizopus stolonifera* by Ochoa-Velasco et al. [35], and is defined as:

(1) $$A=A_{max}*exp\left[-exp\left(\frac{r_{max}*e}{A_{max}}\left(\lambda -t\right)+1\right)\right]$$

where *A* is the average fungal colony size in the Petri dish (mm) at time *t* (d), *A_max_* is the maximal fungal colony size (mm), *r_max_* is the maximum specific growth rate (1/d), *λ* is the lag time (d), and *e* = exp(1), equal to 2.7183. Additionally, r^2^ was determined to evaluate the goodness-of-fit of the experimental data to the selected modified Gompertz equation. The kinetic parameters for each experiment and mathematical adjustment were determined numerically from the experimental data obtained by non-linear regression using the software Sigma-Plot (version 10.0).

### 2.5. OMSW Pretreatment Assays

For this assay, 12 samples of 30 g of sterilized OMSW (49.7% moisture content) from self-sealing bags refrigerated at −20 °C and without any mechanical pretreatment were added to Erlenmeyer flasks (500 mL) according to conditions defined in Table 1. The OMSW final weight on a wet basis in the flasks after sterilization was 28.8 g. Modified Kirk’s medium (MKM) was used to evaluate the need to add supplementary media to improve the enzymatic activity. This medium is widely used for the growth and stimulation of the ligninolytic activity of WRF [36,37]. Distilled water was added to the OMSW assays to facilitate the comparison among the different culture media, avoiding factors such as differences in the mass transference or total solid concentration that would cover up the differences due to the different assayed substrates. Finally, the flasks were inoculated with five disks (6 mm diameter) of active mycelium from 7-day-old culture plates. The flasks were incubated at 25 °C under static conditions for 24 days. Control flasks without OMSW were evaluated at the same time.

### 2.6. Enzymatic Activity Quantification

Extract recovery and enzymatic activity quantification were performed according to Acevedo et al. [36] and Hermosilla et al. [1]. The crude extracts were collected by filtration through Whatman No. 1 filter paper (pore size 11 μm). Enzymatic activity was periodically monitored every two days for 24 days. The enzyme activity of laccase (Lac), manganese peroxidase (MnP), and manganese-independent peroxidase (MniP) was determined through the 2,6-DMP assay, where the reaction mixture contained 200 μL of 50 mM of sodium malonate (pH 4.5), 50 μL of 20 mM 2,6-DMP, 50 μL of 20 mM MnSO_4_.H_2_O, and 50 μL of supernatant. The reaction was initiated by adding 100 μL of 4 mM H_2_O_2_, and the absorbance of the colored product was measured at 468 nm using a UV-vis spectrophotometer (Thermo Scientific Evolution^TM^ 60S) at 30 °C and corrected for the Lac activity [37]. One MnP activity unit (U) was defined as the amount of enzyme transforming 1 μmol 2,6-DMP per minute at pH 4.5 and 30 °C [37]. Total enzymatic activity of WRF was represented as the sum of MniP, Lac and MnP, which were individually determined at the time.

### 2.7. Microstructure Analysis

Structural changes between untreated and pretreated OMSW were appraised by a confocal laser scanning microscopy (CLSM) and scanning electron microscopy (SEM). For CLSM and SEM, representative samples of untreated OMSW and pretreated with both WRF for days 12 and 24 were taken, respectively. In particular, for CLSM the samples were incubated with two markers in the dark for 30 min at room temperature. The markers used were Calcofluor at 20% *v*/*v* for cell wall staining (blue) ʎ emission/excitation 405/450 nm, and Safranin O at 1% *w*/*v* for lignin staining (green) ʎ emission/excitation 546/590 nm. Then, the samples were centrifuged and washed with PBS 1X, and mounted for visualization on a fluodish plate. The samples were visualized in three fluorescence channels using the FV1000 confocal laser microscope (Olympus, Japan), and then the images were analyzed (including the relative fluorescence unit (RFU) quantification) using the FV10 Olympus var 0.2c software. For scanning electron microscopy (SEM), the samples were dried at 25 °C for 4 days and mounted on an aluminum stub and adhered with double-sided carbon tape. After this, samples were outlined using silver paint. The visualization was carried out within the following parameters: 10 KV, WD 10 mm, BSE (chemical contrast), and UVD (topography) detectors in SEM (Hitachi SU3500, Tokyo, Japan).

## 3. Results and Discussion

### 3.1. Olive Mill Solid Waste Characterization

According to Table 2, OMSW had a lignin, hemicellulose, and cellulose content of 33.4 ± 4%, 45.5 ± 8%, and 35.2 ± 3% of the total weight of OMSW, respectively. These values are within the ranges reported in the literature for two-phase OMSW [38]. The organic carbon content of OMSW for the study variety Arbequina is within the range reported in other studies, between 20.5 to 58.5% [26,38]. The determined C/N ratio for the used OMSW was 48.4/0.84 (Table 2). In fact, the much higher concentration of carbon compared to nitrogen is a feature of OMSW that has also been defined as limiting the biological treatment of this biomass with supplementation with a co-substrate being recommended [39,40].

OMSW presented a pH value of around 5.0–5.1, which is within the desirable range for enzyme activity. For example, Bustamante et al. [41] determined that enzyme activity for *A. discolor* in modified Kirk’s medium (MKM) at 25 °C incubation was enhanced at a pH between 5 and 6. On the other hand, some metals from OMSW were determined, giving values for Fe (<13 mg/kg), Mn (<140 mg/kg), and Cu (8.48 mg/kg). The presence of copper and manganese is important due to their role as inducers. Jain et al. [42] showed that with copper addition in the culture media, the laccase activity can be increased up to eight times over culture media without this metal. In the case of Mn, enhancement of MnP production and stimulation of enzymatic activity by WRF has been observed [36,43], showing that expression of MnP in the fungal cultures is dependent on Mn. In addition, Papinutti and Forchiassin [44] indicated that specific MnP activity increases with increasing concentrations of Mn^2+^, reaching a maximum, from which point the activity decreases.

Other analyses have shown a total polyphenol content equivalent to 1.49 g/kg. Compared to other reports, this value is in the low range of total polyphenol content that varies between 1.4 and 10.7 g/kg [16]. OMSW has an antioxidant capacity of 214.4 mg Trolox/L (Table 2). In addition, a phenolic profile analysis was carried out, identifying phenolic acids and flavonols, such as catechin, epicatechin, quercetin, and kaempferol, as shown in Table 2.

### 3.2. Qualitative Detection of Lignocellulolytic Enzymes

The qualitative assay to identify the ligninolytic enzymatic potential of *A. discolor* and *S. hirsutum* was carried out for 14 days. The tested strains of WRF showed enzymatic activities. Specifically, the extent of decoloration with PDA+RBBR resulted in a decoloration ratio between 67 and 90 mm for *A. discolor* (Supplementary Figure S1), and from 22 mm and 45 mm for *S. hirsutum* (Table 3). However, both strains showed a similar activity for PDA+ABTS, resulting in a coloration diameter ranging from 67 to 90 mm. Similar results were published by Tortella et al. [45] for these strains, which indicated that the fungal strains maintained their enzymatic potentials after subcultures since they were isolated. Both strains were selected for further assays in OMSW because they produced the highest reaction intensity with all enzymatic indicators in the media tested, which indicates that they have enzymatic mechanisms to degrade lignocellulosic components of OMSW.

### 3.3. Mycelial Growth Assays

**Figure 1:**
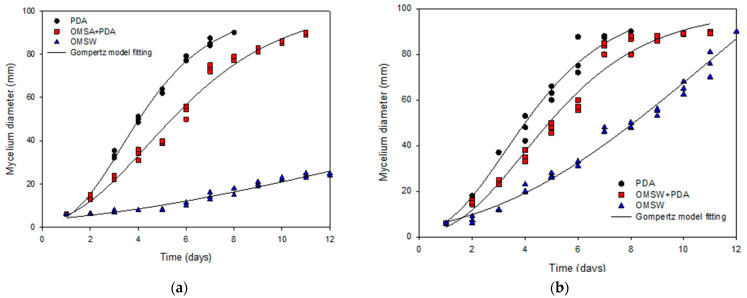
Evolution of fungal growth, equivalent diameters: (**a**) *A. discolor*; (**b**) *S. hirsutum*.

Once the enzymatic activity of the selected WRF strains had been corroborated, the suitability of OMSW as substrate was studied. Figure 1 shows the mycelial growth for *A. discolor* (A) and *S. hirsutum* (B) as a function of culture time using PDA, OMSW, and PDA+OMSW as the substrate. *A. discolor* and *S. hirsutum* showed higher growth using PDA followed by PDA+OMSW and OMSW. When the two strains were cultivated in PDA and PDA+OMSW, shorter latency states (lag phases) were observed than when the OMSW was used as the substrate (Table 4). This behavior may be due to the easier biodegradability of the PDA, which is composed of simple sugars and nitrogen sources more accessible for the fungus than OMSW [46]. In particular, *A. discolor* showed growth from day 6, reaching a maximum mycelial diameter of 20 mm in OMSW, while *S. hirsutum* left the latency state on day 2, reaching a maximum mycelial diameter of 90 mm (Figure 1). In detail, Table 4 shows the mycelial growth rate (mm/d) determined by fitting the modified Gompertz model. In every case, the correlation coefficient (r^2^) values were greater than 0.95, evidencing a statistically significant relationship. However, the value obtained for the maximum mycelial diameter for *S. hirsutum* in OMSW may be overestimated by the proposed model adjustment due to the more complex shape of the curve (Figure 1b, Table 4). As expected, in both strains the highest mycelial growth rate was achieved using PDA, followed by PDA+OMSW and OMSW (Table 4), probably due to the readily biodegradable compounds provided by the PDA [42]. When the substrate was OMSW, *A. discolor* showed a mycelial growth rate of 2.5 ± 0.5 mm/d, while *S. hirsutum* reached 9.7 ± 0.7 mm/d. Although both strains were able to grow in OMSW, the higher mycelial growth rate of *S. hirsutum* compared to *A. discolor* indicated a better adaptability of *S. hirsutum* to grow in this substrate.

### 3.4. Enzymatic Extract Production Using OMSW

**Figure 2:**
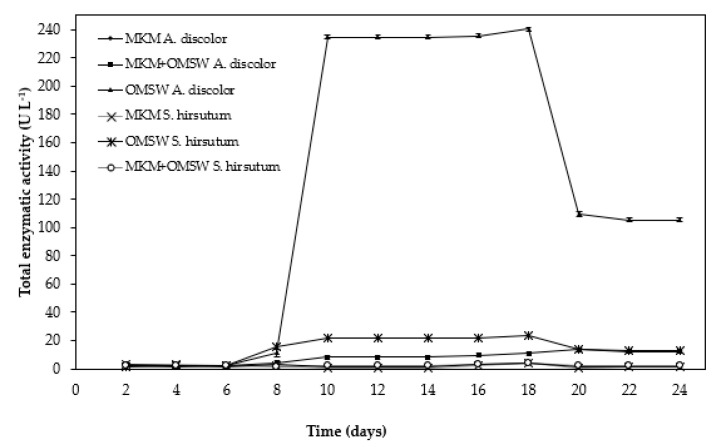
Ligninolytic enzyme activity (represented as sum of Lac, MnP, and MniP), as a function of time using olive mill solid waste (OMSW), OMSW + modified Kirk’s medium (MKM) and modified Kirk’s medium as a substrate for *A. discolor* and *S. hirsutum*.

Figure 2 shows the total activity of the ligninolytic enzymes (represented as the sum of Lac, MnP, and MniP) as a function of time for both strains studied. *A. discolor* was able to produce ligninolytic enzymes in the presence of OMSW, regardless of the supplementation with modified Kirk’s medium. Specifically, *A. discolor* cultivated in OMSW supplemented and not supplemented with modified Kirk’s medium (MKM) reached maximum enzymatic activity as from day 10. By contrast, Shalchli et al. [28] reported maximum enzymatic activity at 15 days using *A. discolor* cultivated in potato peel waste. The same maximum ligninolytic enzyme produced by *Ganoderma lobatum* using wheat straw was obtained after 40 days of incubation [1]. The time difference may be due to the different composition of the substrates used in each study, since the OMSW presented a lignocellulosic content of 33.6% (Table 2), markedly lower than the other reported substrates, e.g., wheat straw contains around 35–45% cellulose, 20–30% hemicellulose, and around 15% lignin [47]. On the other hand, *S. hirsutum* (Figure 2) showed ligninolytic enzyme activity using OMSW, but not for OMSW supplemented with Kirk’s medium or with Kirk’s medium alone. Similarly to *A. discolor*, *S. hirsutum* also showed an increase in enzymatic activity at around days 8–10 (Figure 2).

**Figure 3:**
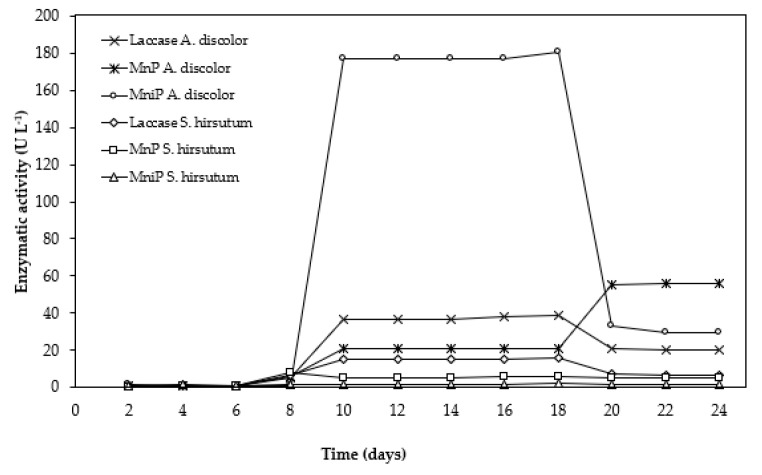
Lac, MnP, and MniP activity variation as a function of culture time using OMSW as the substrate for both strains studied; *A. discolor* and *S. hirsutum*.

Comparing the three conditions studied (OMSW, OMSW+MKM, and MKM), both strains produced the highest amount of ligninolytic enzymes using OMSW, reaching 234.8 U/L for *A. discolor* and 22.1 U/L for *S. hirsutum* on day 10 (Figure 2). By contrast, no strain produced enzymes using MKM. This behavior was likely a consequence of the lignocellulosic waste acting as a support for WRF growth, providing conditions similar to their natural habitat and possibly containing substances that promote the production of the desired enzymes [48,49]. The most likely reason for the increase in their production is the presence of phenols such as ferulic, caffeic, coumaric, and chlorogenic acids [50,51]. Furthermore, the fungal strains would not be pushed to degrade the OMSW to obtain energy in the presence of the MKM due to the availability of enough easily biodegradable soluble organic matter, making the metabolic cost of producing ligninolytic enzymes unnecessary for the fungus. Therefore, although the fungal growth was lower for both strains when cultured in OMSW (Figure 1, Table 4), this condition was the optimal to maximize the enzymatic activity (Figure 3). In addition, white-rot fungi have the ability to secrete extracellular nonspecific ligninolytic enzymes during their secondary metabolism, mainly triggered by the exhaustion of nutrients such as nitrogen [52,53,54,55], carbon [52,54] or sulfur [52]. Thus, when growing on a biomass such as OMSW, there is a period of 8 days that corresponds to fungi primary metabolism, which explains the low enzyme activity found in this work. Then, limited growth conditions are generated at 8 days (Figure 2) due to the restricted availability of nutrients and possibly to the presence of copper in the substrate (8.5 mg/kg) (Table 2) that can limit fungal growth [16]. These conditions allow activation of the extracellular enzymatic machinery of the fungi, and, consequently, the fungi obtain compounds that can be assimilated for fungal nutrition [52,53,54,55]. These results are in agreement with those obtained by Gassara et al. [56], who evaluated the ligninolytic enzyme activity using apple pomace in liquid fermentation. On the other hand, OMSW contains Mn (<13 mg/kg) (Table 2), which may have functioned as an enzyme inducer. In this sense, studies carried out by Gill and Arora, [43] Acevedo et al. [36] Salvachúa et al. [12] and Van Kuijk et al. [5] who used Mn to enhance MnP production and stimulate enzymatic activity by the white-rot fungi, showed that the presence of MnP in the fungal cultures is MN-dependent.

A different case occurs when MKM is used because it is a medium rich in nutrients [57], where the main carbon source is glucose, which, being easily assimilated, maintains the nutritional conditions for the fungus over time, and this condition limits or prolongs the activation of the ligninolytic enzyme. In the results, it is noted that low enzymatic activity can be associated with the previously mentioned conditions. However, it may be possible that with more evaluation time (up to 24 days), the enzymatic production will increase due to the limited nutrient conditions in the culture media.

Figure 3 shows the individual enzyme activity for Lac, MnP, and MniP using *A. discolor* and *S. hirsutum* cultivated in OMSW. *A. discolor* was able to produce the highest amount of MniP, followed by Lac and MnP, reaching a maximum of 176.7, 36.8, and 21.2 U/L, respectively, for each enzyme at day 10, i.e., MniP was produced by *A. discolor* three and eight times more than MnP and Lac, respectively. *S. hirsutum* reported higher Lac productivity over MnP and MniP, reaching 15.3, 5.2, and 1.5 U/L, respectively. The higher presence of MniP may be related to its high selectivity in the degradation of lignin, and also due to the low concentration of manganese in the OMSW (<13 mg/kg OMSW, Table 3), which would have limited the MnP activity in favor of the MniP activity [58,59].

The increase in the activity of MniP and MnP enzymes was previously reported by Reina et al. [60], who described an increase in peroxidase activity from 0% to 19% in 14 days for *Auricularia auricula-judae, Bjerkandera adusta,* and *Coprinellus radians* during the solid-state fermentation of olive mill residues. Likewise, the presence of phenolic compounds from the OMSW would have induced the production of Lac and MnP [60,61]. Although Lac activity was detected, its low production may be due to the C/N ratio of the OMSW, i.e., 60 (Table 2), since some experimental works suggested that substrates with a C/N ratio of less than 16 are the most suitable condition for Lac production [48]. Additionally, substrates with a ratio greater than 40 can strongly limit Lac production by WRF [57,58].

Although the results obtained are interesting, the Lac and MnP values reported in the present work were low compared to 600 and 163 U/L of Lac and MnP, respectively, produced at day 15 for *A. discolor* using potato peel waste as the substrate, reported by Schalchli et al. [28]. However, further research on strategies for enhancing the enzymatic activity would be interesting, considering that culture supplementation with agroindustrial wastes rich in lignin compounds such as OMSW can generate many inducers during fermentation processes, significantly improve the potential of synergic of inducers-enzymes for enzyme extract production, degradation of recalcitrant organic pollutants, removal of recalcitrant lignin, and transformation of its structural polysaccharides into fermentable sugars [62,63].

### 3.5. OMSW Phenol Removal Using A. discolor and S. hirsutum

**Figure 4:**
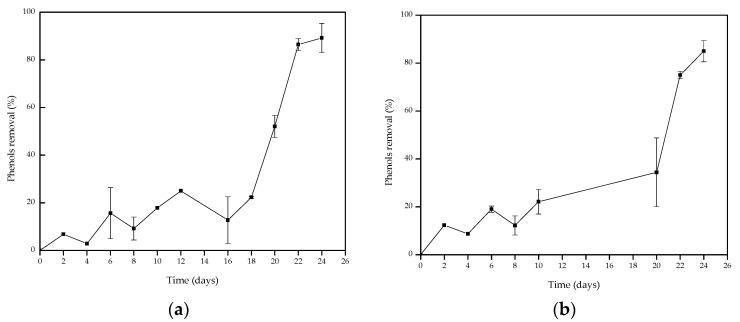
Phenol removal as a function of culture time using OMSW as substrate for both strains studied: *A. discolor* (**a**) *S. hirsutum* (**b**).

The potential of *A. discolor* and *S. hirsutum* in the phenolic detoxification of OMSW was evaluated by monitoring the phenol removal percentage throughout the experimental period (Figure 4). As can be seen, both strains were highly effective at removing phenolic compounds, reaching similar values above 80% at day 24. The highest phenol removal efficiencies being reached at the end of the experimental period is related to the release of new phenolic compounds from complex lignocellulosic structures during the OMSW degradation [64]. The phenol removal efficiencies achieved in the present work were slightly higher than those reported by other authors and were also achieved in a shorter time. In particular, Reina et al. [60] described an efficiency in phenol compounds removal between 60 to 80% for *Auricularia auricula-judae*, *Bjerkandera adusta*, and *Coprinellus radians* after 28 days of OMSW pretreatment. On the other hand, Sampedro et al. [26] found that in OMSW pretreated by *Coriolopsis rigida* the phenolic compound removal was about 36% after 14 days of incubation. By contrast, the phenolic compounds removal was higher than 80% in OMSW pretreated for 140 days, the highest removal percentage being attained with other WRF as *Phlebia radiata* (95.8%), *C. rigida* (89.2%) and *Pycnoporus cinnabarinus* (88.7%).

The phenolic compound removal achieved in the present study stands out over other pretreatments previously proposed for OMSW; for example, some thermal pretreatments have achieved between 58 and 70% removal of phenolic compounds from OMSW. Thus, Serrano et al. [24] found that using a thermal pretreatment with vapor injection at 170 °C for 60 min allowed the separation of OMSW into two phases, solid and liquid, and subsequent phenolic compound removal of 69.5%. Another study by Serrano et al. [65] demonstrated that applying a steam explosion pretreatment with a maximal operating pressure of 42 kg/cm^2^ for 5 min at 200 °C prior to a rapid decompression (explosion) and then centrifugation at 4700× *g* for solid and liquid separation achieved a phenolic compound removal of 58.3%. However, these pretreatments were composed of more stages and high energy requirements compared to the treatment proposed in this study, and therefore, the proposed fungal pretreatments using *A. discolor* and *S. hirsutum* would be a suitable strategy for the detoxification of OMSW in soft conditions (lower temperature (24 °C), not adding other nutrients or agitation systems), allowing the subsequent valorization of this biomass without a high energy cost.

### 3.6. Microstructure Analysis

**Figure 5:**
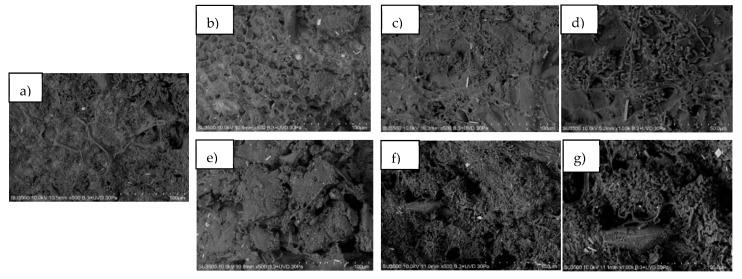
SEM images of: (**a**) untreated OMSW; (**b**) pretreated OMSW with *A. discolor* at 12 (**b**) and 24 (**c**,**d**) days; pretreated OMSW with *S. hirsutum* at 12 (**e**) and 24 (**f**,**g**) days.

The morphology of the untreated OMSW and fungal pretreated OMSW at 12 and 24 days of degradation is shown in Figure 5. The untreated OMSW (Figure 5a) shows clearly defined fibers. By contrast, in Figure 5b–g, fibers are not distinguished. Particularly in Figure 5b, for day 12 of treatment with *A. discolor*, a greater number of pores are observed than in the untreated OMSW. These results are according to Mishra et al. [62], who reported the formation of holes and crevices on the biomass surfaces in pretreated sweet sorghum bagasse.

**Figure 6:**
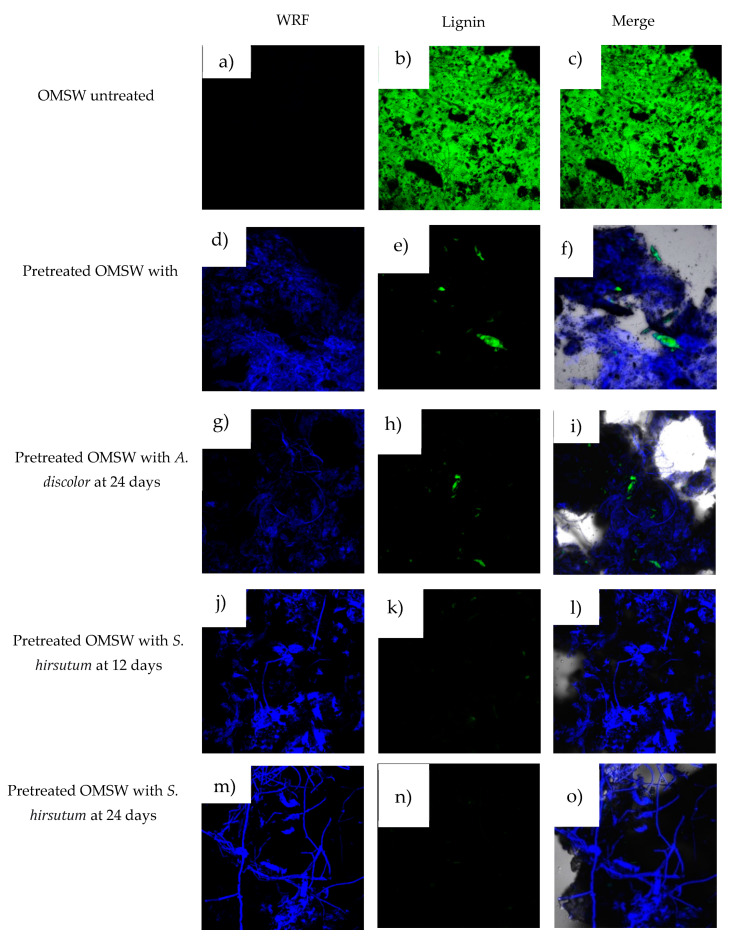
Confocal images of untreated OMSW (**a**–**c**); pretreated OMSW with *A. discolor* at 12 (**d**–**f**) and 24 (**g**–**i**) days; pretreated OMSW with *S. hirsutum* at 12 (**j**–**l**) and 24 (**m**–**o**) days.

The lignocellulosic structure of the untreated OMSW and pretreated OMSW at 12 and 24 days of degradation is shown in Figure 6. In particular, Figure 6a–c shows the untreated OMSW; the green staining is selective for lignin and blue for WRF, clearly showing the OMSW lignin composition. Figure 6d–i,j–o shows the OMSW after 12 and 24 days of treatment using *A. discolor* and *S. hirsutum*, respectively. It can be seen as green in untreated biomass, which could be due to lignin degradation using both WRF. On the other hand, blue indicates the growth of both fungi in the OMSW.

The relative fluorescence units (RFU) obtained from the CLSM analysis showed that lignin fluorescence decreased from 3967 RFU for untreated OMSW to 235 and 221 RFU, showing a lignin relative degradation of 94.1% and 94.4% after 24 days of treatment using *A. discolor* and *S. hirsutum,* respectively (Table 5). At the same time, the fungi fluorescence increased from 291 to 3548 and 3423 at 12 and 24 treatment days for *A. discolor* and *S. hirsutum*, indicating WRF growth while lignin is degrading.

Although the present work did not exhibit high ligninolytic enzyme activity (see point 3.4) compared to results that have been reported using *A. discolor* for pretreatment of wheat straw [1] and potato peel [28], our results demonstrate that the enzyme activities obtained are sufficient to remove phenolic compound (point 3.3) and lignin degradation. Despite *S. hirsutum* showing a low ligninolytic enzymatic activity compared to *A. discolor*, both fungi demonstrated the ability to remove phenolic compounds and lignin degradation from OMSW, thereby proving for the first time the applications of this strain for OMSW pretreatment. To the best of our knowledge, *S. hirsutum* has never been used for pretreatment of OMSW. As shown in Table 5, there are no reports about lignin degradation in OMSW using WRF.

Further characterization (such as glucose, fatty acids, chemical oxygen demand) of OMSW pretreated with WRF are needed since it could subsequently be fermented into ethanol or used for the production of other chemicals (for example biogas) in a biorefinery concept, it being feasible to apply a biological pretreatment using *A. discolor* or *S. hirsutum*. For example, Hermosilla et al. [19] reported that *Ganoderma lobatum* showed a 50.3% lignin degradation after 40 days. This fungal pretreatment significantly increased the amount of glucose released from wheat straw, reaching 97.4 mg of glucose/g of wheat straw.

The results of this work demonstrate that *A. discolor* and *S. hirsutum* have the phenolic compound removal and lignin degradative abilities for a pretreatment of OMSW, applying WRF directly without the need to add nutrients, exogenous carbon source, or use agitation systems. Moreover, the main issues found are associated with: (1) high reaction time (24 days) compared with thermal treatments (no more than 1 h), and (2) water addition (OMSW/water of 1:3.3) (Table 5).

However, OMSW is seasonal waste produced one season per year, being stored for a long time [68]. Therefore, the low reaction might not be a problem since the application of the proposed pretreatment could be carried out in situ. In terms of the water addition, the necessary optimization will be addressed in future research.

## 4. Conclusions

The WRF *A. discolor* and *S. hirsutum* were able to grow under static conditions using OMSW as the sole substrate without supplementing other nutrients, reaching more than 80% of total phenol removal, with clear evidence of lignin degradation after 24 days. These results indicate that it could be possible to design an in situ pretreatment of the valorization of OMSW, avoiding complex systems or transportation. In this sense, future research under non-sterile conditions is needed to evaluate the competition of WRF with other microorganisms present in the OMSW. In addition, water use must be optimized in order to minimize it.

## Figures and Tables

**Table 1:** Culture conditions for enzymatic extract production using OMSW.

Strain	Culture Media
*S. hirsutum*	100 mL MKM
*A. discolor*	100 mL MKM
*S. hirsutum*	100 mL MKM + 30 g OMSW
*A. discolor*	100 mL MKM + 30 g OMSW
*S. hirsutum*	100 mL distilled water + 30 g OMSW
*A. discolor*	100 mL distilled water + 30 g OMSW

OMSW: Olive mill solid waste; MKM: Modified Kirk’s medium.

**Table 2:** Physicochemical characteristics of olive mill solid waste.

Characteristics	Value
α-cellulose (% *w*/*w*)	35.2 ± 3
Lignin (% *w*/*w*)	33.4 ± 4
Hemicellulose (% *w*/*w*)	45.5 ± 8
Elemental analysis (C/N) (% *w*/*w*)	48.4/0.84
Ash (% *w*/*w*)	2.96
Total nitrogen (mg/kg)	134.2
Nitrate (mg/kg)	<1.0
Nitrite (mg/kg)	<0.6
Total copper (mg/kg)	8.5
Total iron (mg/kg)	<140.0
Total manganese (mg/kg)	<13.0
Chemical oxygen demand (mg O_2_/L)	87.9
Total solids (% *w*/*w*)	38.5
Volatile solids (% of TS)	89.7
pH	5.08
Total polyphenols (mg GA/100 g)	149.0
Antioxidant capacity (mg Trolox/L)	214.4
Gallic acid hexoside (mg/100 g)	0.9
Gallic acid protocatechuic acid hexoside (mg/100 g)	0.5
Hexoside ferulic acid (mg/100 g)	0.3
Cautaric acid (mg/100 g)	1.0
Caftaric acid (mg/100 g)	0.9
Catechin (mg/100 g)	1.6
Epicatechin (mg/100 g)	1.9
Quercetin-3-rutinoside (mg/100 g)	1.3
Quercetin-3-hexoside (mg/100 g)	1.7
Quercetin-3-glucuronide (mg/100 g)	2.0
Kaempferol-3-glucoside (mg/100 g)	3.5
Kaempferol-3-hexoside (mg/100 g)	10.2
Lipids (% *w*/*w*)	13.23

**Table 3:** Qualitative detection by RBBR decoloration and ABTS coloration at evaluation day 14.

Strain	PDA+RBBR (Decoloration)	PDA+ABTS (Coloration)
*A. discolor*	++++	++++
*S. hirsutum*	++	++++

Decoloration and coloration scale at 14 days of cultivation. ++: diameter > 22 mm and ≤ 45 mm, ++++: diameter > 67 mm and ≤ 90 mm and 0: no effect.

**Table 4:** Kinetics results of the modified Gompertz model applied to the fungal growth studied.

Culture Conditions	Maximum Mycelial Diameter (mm) (A_max_)	Mycelial Growth Rate (mm/day) (r_max_)	Lag Phase (d) (λ)	e	R^2^
PDA medium					
*A. discolor*	100 ± 2	17.9 ± 0.5	1.2 ± 0.1	2.7183	0.9968
*S. hirsutum*	102 ± 5	16.8 ± 1	1.0 ± 0.2	2.7183	0.9838
OMSW					
*A. discolor*	78 ± 39	2.5 ± 0.5	1.6 ± 1.9	2.7183	0.9585
*S. hirsutum*	212 ± 46	9.7 ± 0.7	3.0 ± 0.6	2.7183	0.9857
PDA and OMSW					
*A. discolor*	103 ± 4	12.1 ± 0.5	1.3 ± 0.1	2.7183	0.9864
*S. hirsutum*	100 ± 3	14.3 ± 0.9	1.4 ± 0.2	2.7183	0.9793

PDA: potato dextrose agar; OMSW: olive mill solid waste.

**Table 5:** Comparison with similar works.

Strain	Phenolic Compounds Removal (%)	Reaction Time (days)	Specific Conditions		OMSW (g)/Water (mL) Proportion	References
			Temperature (°C)	Others		
*Phanerochaete flavido-alba*	70	60	30	Aeration: sterile O_2_ (3 L/min for 1 min) every 24 h	1:0.3	[66]
*P. radiata*	95.8	140	30	-	1:0.1	[67]
*C. rigida*	89.2					
*P. cinnabarinus*	88.7					
*Phlebia* *sp.*	85	30	28	Immobilization in Polyurethane sponge	1:0.1	[26]
*Panus tigrinus*	36					
*Phlebia* *sp.*	43					
*A. auricula-judae*	60	28	24	-	1:4.5	[60]
*B. adusta*	80					
*C. radians*	75					
*A discolor*	90	24	25	-	1:3.3	This work
*S. hirsutum*	85					

-: not reported.

## Data Availability

Data is contained within the article or Supplementary Material.

## Supplementary Materials

The following figures and tables are available online at https://www.mdpi.com/article/10.3390/foods11111587/s1, Figure S1: Qualitative detection of lignocellulosic enzymes. (a) control with ABTS and PDA, (b) *A. discolor* in PDA with ABTS day 1, (c) *A. discolor* in PDA with ABTS day 14, (d) *S. hirsutum* in PDA with ABTS day 1, (e) *S. hirsutum* in PDA with ABTS day 14, (f) control with RBBR and PDA, (g) *A. discolor* in PDA with RBBR day 1, (h) *A. discolor* in PDA with RBBR day 14, (i) *S. hirsutum* in PDA with RBBR day 1, (j) *S. hirsutum* in PDA with RBBR day 14.
