# Supplementary material for: Use of Anthracophyllum discolor and Stereum hirsutum as a Suitable Strategy for Delignification and Phenolic Removal of Olive Mill Solid Waste
Source: Foods. 2022 May 28;11(11):1587. doi: 10.3390/foods11111587 (PMC9180551; doi:10.3390/foods11111587)
Supplement: Supplementary file 1 [file foods-11-01587-s001.zip › foods-1668829-supplementary.pdf]

Supplementary material

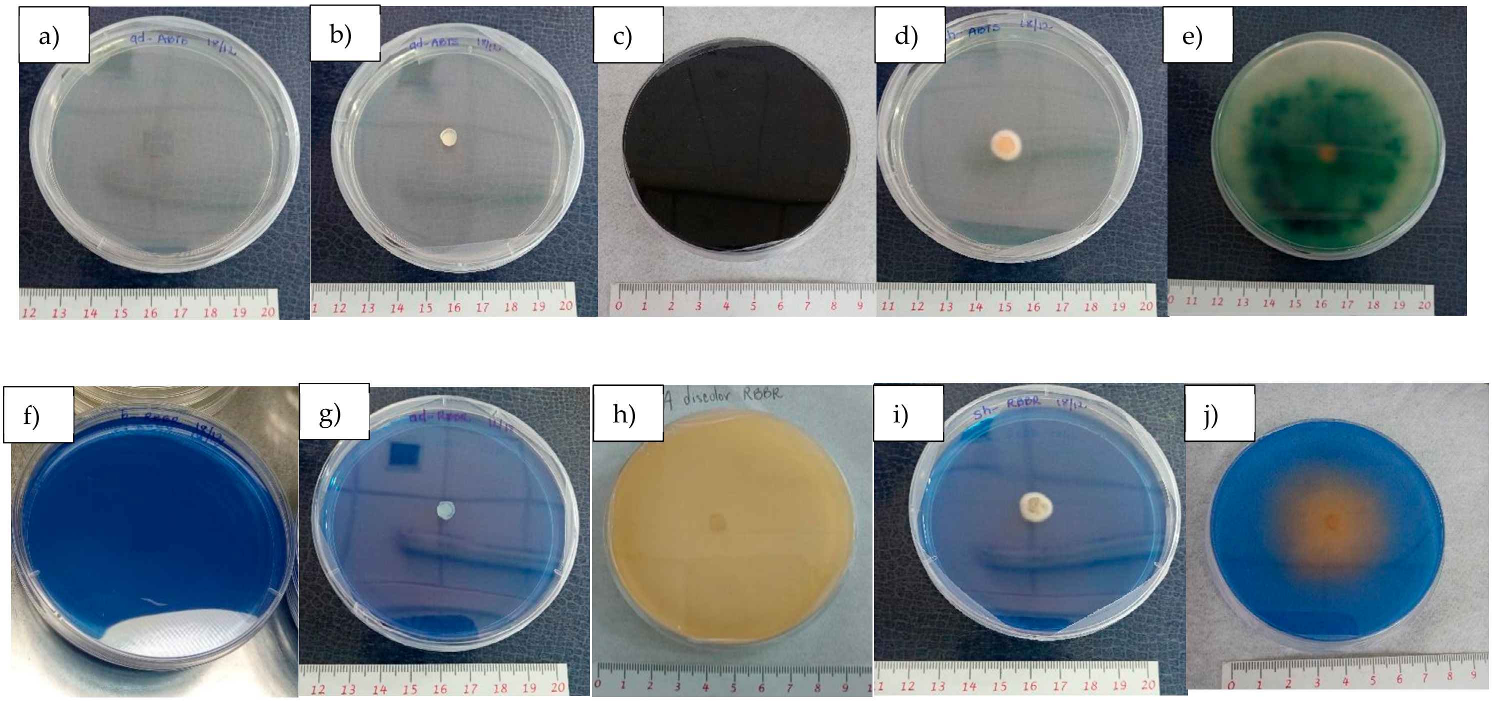

Figure S1. Qualitative detection of lignocellulosic enzymes. (a) control with ABTS and PDA, (b) *A. discolor* in PDA with ABTS day 1, (c) *A. discolor* in PDA with ABTS day 14, (d) *S. hirsutum* in PDA with ABTS day 1, (e) *S. hirsutum* in PDA with ABTS day 14, (f) control with RBBR and PDA, (g) *A. discolor* in PDA with RBBR day 1, (h) *A. discolor* in PDA with RBBR day 14, (i) *S. hirsutum* in PDA with RBBR day 1, (j) *S. hirsutum* in PDA with RBBR day 14.
